# Therapeutics

**Published:** 1904-06

**Authors:** 


### PROGRESS IN MEDICAL SCIENCE.

### Therapeutics.

### CARDIAC TONICS IN PNEUMONIA.

D. B. Lees, M.D., F.R.C.P., (*British Med. Jour.,* November 28, 1903), in the second Harveian Lecture, considers that it is generally advisable to begin the use of heart tonics about the third or fourth day of the disease and that they ought to be given, if at all, somewhat freely.

Of these drugs strychnine is probably the most useful and should be given hypodermatically.

Atropine by subcutaneous injection is also very serviceable in children, but not so useful for adults because they suffer much more than children from the dryness of throat and other unpleasant effects of belladonna. In children large doses of this drug will cause chiefly flushing of skin which is of no importance.

Oxygen by inhalation assists the aeration of blood in the lungs, and thus improves the quality of the blood supplied to the cardiac muscle. It is therefore truly a cardiac tonic. Its use should be begun as soon as cyanosis is definite, and should be continued for five minutes every hour, whether the patient is awake or asleep. It can be given without disturbing him in the least.

Oxygen is certainly a most valuable remedy, and ranks with strychnine in the treatment of pneumonia. But neither strychnine nor oxygen, nor both together, will often save life if the right auricle be not relieved. After a bleeding they are powerful remedies; without removal of blood they often fail, and almost necessarily. It is good to maintain the strength of the cardiac muscle; it is still better to diminish its labor. It is best to do both.

Digitalis will not always reduce the frequency of the pulse in pneumonia, especially when the temperature is high. It is most likely to be of service after relief of the right heart, when the fever is moderate and the pulse still remains weak and frequent.

Ammonium carbonate may be given when there is evidence of much secretion in the bronchial tubes.

Alcohol, though called a "stimulant," has not much title to be considered a cardiac tonic. It is essentially a vasomotor depressant, and as such may help the heart indirectly when the tension is high. There is also sometimes a temporary increase in the strength of the pulse after the administration of a moderate dose, probably due to increased blood supply to the cardiac muscle, through relaxation of coronary arterioles. It is therefore possible that repeated small doses may be of service in pneumonia, but the large doses sometimes advised are likely to do more harm than good. To imagine that brandy can "support" the heart when the right side is becoming paralysed from overdistension is absurd. In such a case the only satisfactory cardiac tonic is a venesection.—*Review of Reviews.*

---

### TYPHOID FEVER.

Egbert LeFevre, (*Medical News,* January 2, 1904), states that against the action of the typhoid toxin on the heart and nervous system, two agents have been especially advocated, viz.: strychnine and alcohol. At the present time strychnine is the more commonly used, and he strongly dissents from the present plan of giving strychnine in heroic doses as soon as typhoid fever is diagnosticated. It is not unusual to find patients both in hospital and private practice, who have gone through the first week or ten days of typhoid fever so freely drugged with strychnine that their reflexes were exaggerated. In these cases much of the restlessness and distress, supposed to be due to the disease, is actually caused by medication. He believes that patients who are early stimulated to the full physiologic limit by strychnine make a slower convalescence, and that they are more prone to vasomotor disturbance and nervous depression during this stage. Strychnine should be reserved until the condition of the reflexes, both cardiac and spinal, show that the disease has begun to affect the nervous centers. The dose should then be regulated to meet the indications. As regards alcohol, he believes that while in the animal and healthy individual it may show no action upon the circulatory system beyond a reflex one, in diseased conditions, especially the infectious diseases, it has a different action. We are all familiar with the fact that large doses can be given in febrile cases without symptoms of intoxication being induced, or the odor of alcohol appearing on the breath, and that under its use the nervous system is quieted and the heart beat slowed. Whether in health alcohol acts as a food is not germane to the discussion; certainly in typhoid fever alcohol acts both as a stimulant and a food, and when the diet is deficient, as when a milk diet alone is given, nutrition is improved and emaciation retarded.—*Cleveland Medical Journal.*

---

### THE USE OF ARSENIC.

POPE (*International Medical Annual*), lays down the following rules: see that the tongue is clean. Put the patient on a bland, easily digested diet. Use the drug in a much diluted form and in the same dilution throughout. Starting with 2½ minims of Fowler's solution to the ounce of water, thrice daily, give two ounces of the same strength next day; three ounces the day after, and so on as long as no unpleasant symptoms occur. Small doses may be given after meals, large doses during meals. In adults the initial dose may be two or three ounces of the mixture thrice daily. Do not discontinue at the first attack of vomiting. If vomiting persists discontinue drug for 24 hours, and then resume with the same dose. Examine daily for toxic symptoms.—*Denver Medical Times.*

---

### NEURALGIA.

C. H. FRAZER, (*American Jour. of Med. Sciences,*) December, 1903,) states that trifacial neuralgia is probably in about 20 per cent. of the cases amenable to treatment other than operative. In some cases the disease has a tendency to run its course, usually reaching its height in five or six years, and then exhibiting a tendency to spontaneous cure. The medicinal treatment consists, first, in the removal of all predisposing causes, as malaria, anemia, exhaustion, or any peripheral irritation, such as a carious tooth, or antral disease. Secondly, the use of drugs, and the drug which is, *par excellence,* the most efficacious, especially so in the exhausted and anemic state, is strychnine. In cases of but one or two years' standing, strychnine properly administered will arrest or control the disease almost invariably. In order to obtain this result the drug must be administered in heroic doses, and the patient must be kept under the closest observation, and should be confined to bed. The remedy is administered hypodermically once daily, in gradually ascending doses, until at the expiration of two weeks the physiologic limit is reached. Thus beginning with one-thirtieth of a grain daily the dose may be increased to one-tenth or one-eighth, or higher, and when the maximum dose is reached it should not be given oftener than once on alternate days. After the pain has entirely disappeared the drug should be gradually withdrawn. As adjuvants to this treatment rest is regarded as of the utmost importance, and iodide of potassium and the tincture of chlorid of iron are regarded as more or less helpful. It is perfectly proper and justifiable to give medicinal measures a fair trial for a year at the utmost, and then if the attacks are very frequent, severe and uncontrollable, operative intervention is the only hope of relief.—*Cleveland Med. Jour.*

---

Howard S. Anders, (*Medical News,* March 19, 1904,) states that arsenic is an improver of nutrition, seeming to be a direct stimulant to nutrition by diminishing or checking tissue waste. Indirectly it improves nutrition by increasing the recuperative powers, and some people need arsenic to create a normal neuro-muscular vigor, just as the syphilitic needs the iodids, and the chlorotic, iron. He hesitates in regard to employing it in cases of tuberculosis, although he believes it to be of value in those cases in which fever is absent. He believes it almost a specific in Saint Vitus's dance, and thinks it best administered by beginning with about three drops of Fowler's solution and increasing about two or three drops each successive day. He considers the drug of great importance in the treatment of chronic malaria, anemias, cachexias, and in intermittent malarial neuralgia.—*Cleveland Med. Jour.*

---

FOR SCIATICA.

| | | |
|---|---|---|
| ℞ | Calci glycerophosphatis. | |
| | Magnesii glycerophosphatis . . . āā | 15 grains. |
| | Potassii glycerophosphatis | 4 drams. |
| | Sodii glycerophosphatis | 1½ drams. |
| | Aqua destil | 6 drams. |

M.—Ft. sol. Sig.—Inject one drachm (4 gm.) into seat of the pain once daily.—*Medical Review of Reviews.*

CHRONIC RHEUMATISM.

| | | |
|---|---|---|
| ℞ | Sodium iodid | 2 drams. |
| | Wine of colchicum root | 4 drams. |
| | Sodium salicylate | 3 drams. |
| | Ammoniated tincture of guaiacum | 2 ounces. |
| | Compound syrup of sarsaparilla, sufficient to make | 6 fluid ounces. |

Dessertspoonful three times daily.—*Merck's Archives.*

| | | |
|---|---|---|
| ℞ | Thiocol | 30 grains. |
| | Sodium benzoate | 30 grains. |
| | Tincture of aconite | 20 drops. |
| | Syrup of poppy | 10 drams. |
| | Cherry-laurel water | 2½ drams. |
| | Syrup of senega, sufficient to make | 5 ounces. |

Tablespoonful three times daily.—*La Presse Med.*

